# Problems and Promises of Health Technologies: The Role of Early Health Economic Modeling

**DOI:** 10.15171/ijhpm.2019.36

**Published:** 2019-06-22

**Authors:** Janneke P.C. Grutters, Tim Govers, Jorte Nijboer, Marcia Tummers, Gert Jan van der Wilt, Maroeska M. Rovers

**Affiliations:** ^1^Department for Health Evidence, Radboud Institute for Health Sciences, Radboudumc, Nijmegen, The Netherlands.; ^2^Medvalue, Radboudumc, Nijmegen, The Netherlands.; ^3^Department for Health Evidence, Donders Institute for Brain, Cognition and Behaviour, Radboudumc, Nijmegen, The Netherlands.; ^4^Department of Operating Rooms, Radboud Institute for Health Sciences, Radboudumc, Nijmegen, The Netherlands.

**Keywords:** Innovation Policy, Innovation, Health Technology Assessment, Health Economic Modeling, Early Assessment

## Abstract

**Background:** To assess whether early health economic modeling helps to distinguish those healthcare innovations that are potentially cost-effective from those that are not potentially cost-effective. We will also study what information is retrieved from the health economic models to inform further development, research and implementation decisions.

**Methods:** We performed secondary analyses on an existing database of 32 health economic modeling assessments of 30 innovations, performed by our group. First, we explored whether the assessments could distinguish innovations with potential cost-effectiveness from innovations without potential cost-effectiveness. Second, we explored which recommendations were made regarding development, implementation and further research of the innovation.

**Results:** Of the 30 innovations, 1 (3%) was an idea that was not yet being developed and 14 (47%) were under development. Eight (27%) innovations had finished development, and another 7 (23%) innovations were on the market. Although all assessments showed that the innovation had the potential to become cost-effective, due to improved patient outcomes, cost savings or both, differences were found in the magnitude of the potential benefits, and the likelihood of reaching this potential. The assessments informed how the innovation could be further developed or positioned to maximize its cost-effectiveness, and informed further research.

**Conclusion:** The early health economic assessments provided insight in the potential cost-effectiveness of an innovation in its intended context, and the associated uncertainty. None of the assessments resulted in a firm ‘no-go’ recommendation, but recommendations could be provided on further research and development in order to maximize value for money.

## Introduction

Novel health technologies are being developed at a dizzying pace. 3D printing, biomarkers, genomics and robotics are only some examples of innovative trends. Such innovations are widely assumed to be positive in their effects, to the extent that the term ‘innovative’ usually expresses unqualified praise.^1^ Yet, the need to avoid unnecessary innovations and manage the transition for necessary innovations, is among the most important challenges facing health systems today.^2^

Health economic modeling has traditionally been applied to new health technologies as a reimbursement decision tool once the technology had satisfied regulatory approval, and evidence on costs and consequences is available. It can then act as a means to synthesize available evidence and estimate the resulting uncertainty.^3^ However, this might not be the first opportunity in the technology lifecycle for this evaluation to occur.^4^ Over the last 2 decades, there has been an increasing interest in using health economic modeling in earlier stages of technology development, to inform product development, market access, and pricing.^5^ In this so-called early health technology assessment (HTA), which includes early health economic modeling, the focus is often on the ‘commercial viability’ of new technologies.^6-10^ This is deemed to allow companies to stop further development if results suggest that the product is unlikely to become cost-effective. Ijzerman et al refer to this as “fail fast, fail cheap.”^5^

Especially in such early stages, technology can still be developed in many different ways.^2^ Lehoux and colleagues recently published the results of their 5-year qualitative research program examining health innovation processes. They suggest that “early HTA and coverage with evidence development initiatives could provide technology developers with useful input regarding the decisions they make.”^2^ These decisions may transcend the go/no-go decision that is the current focus of early HTA.^5,10,11^ To date, such a larger role for early HTA is not yet formally substantiated with empirical evidence.

Over the past 4 years, through a subsidiary company of our university hospital, we have performed 32 early-stage health economic modeling assessments. The aim of this study is to explore whether these assessments help to distinguish those innovations that are potentially cost-effective from those that are not potentially cost-effective. Additionally, we explore what information is retrieved from the health economic models to inform further development, research and implementation decisions.

## Methods

### Description of the Assessments 

We retrospectively analyzed the first 32 early-stage health economic modeling assessments performed by our group. These assessments related to 30 innovations. Innovations were assessed if they had the aim to improve patient health, and if a stakeholder commissioned a health economic modeling assessment. Two innovations (#16 and #19 in the Table) were assessed twice, exploring different scenarios for the positioning of the innovation in the care pathway. The assessed innovations were aimed at improving healthcare over a wide range of diseases, such as cancer, diabetes, pregnancy-related complications and asthma (Supplementary file 1). They were all non-drug technologies, relating to the screening (n = 5), diagnosis (n = 11) or treatment (n = 14) of patients (Table).

**Table:** Overview of the Assessments, Innovations and Retrieved Information^a^

**Assessment #**	**Type of Innovation**	**Development Phase**	**Clinical Data on Innovation Available?**	**Further Development**	**Further Research**
1	Diagnosis	Pre-market	No	Positioning	Value proposition
2	Treatment	Pre-market	Yes	-	Value proposition
3	Screening	Concept development	No	Positioning	Value proposition
4	Treatment	Concept development	No	Value proposition	Value proposition
5	Treatment	Concept development	No	Positioning	-
6	Diagnosis	Market access	Yes	Positioning	Value proposition
7	Treatment	Market access	No	-	Value proposition
8	Diagnosis	Pre-market	No	-	Value proposition
9	Screening	Concept development	No	Development	Value proposition
10	Diagnosis	Concept development	No	Positioning	Value proposition
11	Treatment	Idea screening	No	Development	-
12	Treatment	Concept development	No	Value proposition	Value proposition
13	Diagnosis	Market access	Yes	Positioning	-
14	Treatment	Pre-market	No	Value proposition	Value proposition
15	Treatment	Concept development	No	Positioning	Value proposition
16a	Screening	Pre-market	Yes	Positioning	Value proposition
16b	*Same innovation as 16a*			Positioning	Value proposition
17	Diagnosis	Concept development	No	Positioning	Usual care
18	Treatment	Concept development	No	Positioning	Value proposition
19a	Diagnosis	Pre-market	Yes	Positioning	-
19b	*Same innovation as 19a*			Positioning	-
20	Diagnosis	Concept development	No	Positioning	Value proposition
21	Screening	Concept development	No	Positioning	Value proposition
22	Treatment	Concept development	No	Positioning	Value proposition
23	Treatment	Market access	Yes	Value proposition	Patient benefit
24	Diagnosis	Market access	Yes	Positioning	Patient benefit
25	Diagnosis	Market access	No	Positioning	Value proposition
26	Treatment	Pre-market	No	Positioning	Value proposition
27	Treatment	Pre-market	No	Positioning	Value proposition
28	Screening	Concept development	No	Positioning	Value proposition
29	Treatment	Market access	No	Value proposition	Value proposition
30	Diagnosis	Concept development	No	Positioning	Value proposition

^a^More detailed information on the innovations and assessments can be found in Supplementary file 1.

In each of the 32 assessments, a deterministic health economic model was built. The models were conceptualized and validated in accordance with the modeling good research practices.^12,13^ Each model was informed and validated through interviews with independent clinical experts and where relevant with other stakeholders. The models were either state transition models or decision trees, or a combination of the 2. Most of the assessments (91%) were commissioned by medical device companies, generally small to medium enterprises. The other 3 assessments were commissioned by clinicians and/or clinical departments within our hospital. The commissioner of the assessment will further be referred to as ‘client.’

In these 32 health economic modeling assessments, evidence from published literature was synthesized to estimate the costs and effects of the current care pathway. If a parameter could not be informed by evidence, estimates were obtained from independent clinical experts and varied in sensitivity analyses. To estimate potential cost-effectiveness, costs were calculated from a Dutch healthcare perspective, unless the client requested a different perspective. Where possible, effectiveness was measured in terms of quality-adjusted life years (QALYs). Costs and effects were discounted in adherence to the guidelines for economic evaluation from the relevant jurisdiction.

Different analyses had been performed to assess the potential value of an innovation, depending on the question of the client and the availability of evidence. First, if the costs and effects of the innovation were unknown, effectiveness gap or headroom analysis was performed to explore the maximum potential value of an innovation.^6^ Here, care as usual is compared with the perfect situation that the innovation could – in theory – achieve. The difference in costs and effects represents an upper bound of the potential cost-effectiveness of the innovation. Second, if the costs of an innovation or its effects were known, threshold analysis was performed.^14^ Here, we searched for the minimum effectiveness that is needed for the innovation to be cost-effective, given its costs. Or, we searched for the maximum costs of the innovation given its effectiveness. Third, scenario analysis was used to develop descriptions of how the future may unfold based on ‘if-then’ propositions, and to evaluate the consequences of these descriptions in terms of costs and effects.^15^ In these scenarios we explored cost-effectiveness of the innovation under different assumptions of costs, effectiveness or for example a different subpopulation or positioning in the care pathway. Fourth, if clinical evidence on costs and effects of the innovation was available, ‘standard’ cost-effectiveness analysis was performed based on this evidence. Additionally, in all assessments uncertainty about care as usual was handled by means of deterministic sensitivity analysis. Since the current study involves a retrospective analysis of completed assessments, no additional analyses of (potential) cost-effectiveness were performed for this study.

### Analysis of the Assessments

First, we determined the development phase of the innovation at the time of assessment. Four development phases were distinguished. First, in the ‘idea screening’ phase the innovation is not yet being developed, but only consists of an innovative idea. Second, in the ‘concept development’ phase a first version of the innovation is being developed. Third, in the ‘pre-market’ phase a product is available, for example for clinical research, but the innovation is not yet on the market. Finally, in the ‘market access’ phase the innovation has entered the market and is used in clinical practice.

Second, from the 32 assessments we retrospectively retrieved what types of decisions were informed, and how. For this purpose we first explored whether the assessments found the innovations to have potential to become cost-effective or not. An innovation had the potential to become cost-effective if it could yield both health benefit and cost savings, or if it had the potential to remain below the relevant cost-effectiveness threshold. For example, most innovations were assessed from a Dutch healthcare perspective and thus compared to the Dutch cost-effectiveness threshold of €20 000 to €80 000 per QALY.^16^ As Chapman et al put it, early health economic modeling can be used to inform developers “to avoid investment in devices that could never be cost-effective.”^11^ If possible, we calculated the percentage of innovations that could never be cost-effective. Second, we explored which other insights were obtained from the assessments. We categorized these into insights for further development and/or implementation, and insights for further research. Where possible, these categories were further specified to identify clusters of specific types of insights. For each of these clusters, percentages and examples were provided.

The assessments were independently analyzed by 2 authors (JG, TG). Discrepancies were resolved in a consensus meeting with a third author (JN).

## Results

### Phases of Development 

**Figure 1:**
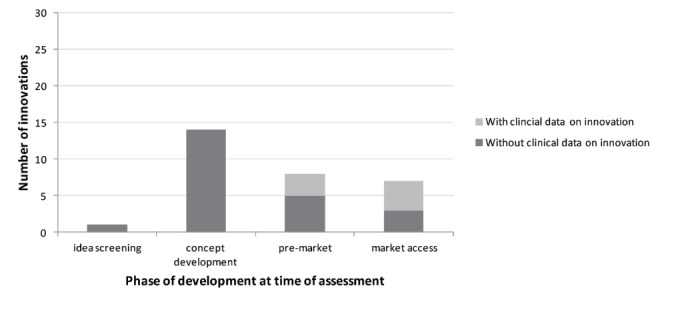
Overview of the Phases of Development at the Time of Assessment.

Of the 30 innovations, 1 (3%) was in the idea screening phase and had not yet started its development (Figure 1).

Here, the room for improvement (headroom) was assessed, accompanied with scenario analyses (Supplementary file 1). This assessment is described in more detail in Supplementary file 2, to illustrate the different types of analyses that were performed. For 14 (47%) innovations that were in the phase of development and not yet studied in patients, headroom, scenario and threshold analyses were performed. In general, multiple types of analyses were combined in one assessment. For example, we explored under which circumstances the innovation could provide value for money (threshold analysis), as well as an analysis to explore what if the innovation had a specific effectiveness (scenario analysis). Another 8 (27%) innovations had finished development and were planning or performing clinical (pilot) studies. For 3 of these innovations, some clinical data on the performance of the innovation was already available. Here, standard (preliminary) cost-effectiveness analyses were performed. For example, data on accuracy of a novel diagnostic test were combined with published literature on the implications of false negative and false positive results. Seven (23%) innovations were in the market access phase. For 4 of these innovations some evidence on its performance was available. Depending on the availability of evidence, (preliminary) cost-effectiveness analyses or threshold/scenario analyses were performed.

### Distinguishing Between Innovations With and Without Potential Cost-Effectiveness

Of the 32 early assessments performed by our group, all showed that the innovation could potentially become cost-effective, due to improved patient outcomes, cost savings or both. Innovations for which we could not estimate the potential benefit in terms of QALYs, were deemed potentially cost-effective because they potentially resulted in both cost savings and health gain. Hence, for none of the innovations we could conclude that it could never become cost-effective, and thus none of the assessments resulted in a firm no-go recommendation.

We did find that the assessments were helpful in gaining insight in the potential cost-effectiveness of the innovation in its intended context. For example, we assessed the potential cost-effectiveness of an innovative device (#26) that aimed to prevent side effects caused by first-line treatment. Patients with side effects generally stop treatment or receive a lower dose, both resulting in a lower total treatment dose. Reducing side effects would therefore increase the dosage of this first-line treatment to the total dose that was recommended in the guideline, resulting in an increase in costs. Very recent studies however showed that on average this increase to the recommended total dose for these specific patients did not increase efficacy or improve health. Thus, the innovation could yield some cost reduction and health benefit due to a reduction in side effects, but this would come at the high cost of increased dose of expensive first-line treatment. Although this might be deemed ‘unfair,’ as the innovation cannot be blamed for the fact that in these patients first-line treatment does not provide value for money, the (potential) cost-effectiveness of an innovation is always dependent on its context. Therefore, it is important for developers to understand this context and to know in an early stage of development what the potential cost-effectiveness of their innovation might be, and which factors influence this cost-effectiveness. Of course, health economic modeling is only one tool to understand this context, and interviews with stakeholders to build and validate the model are essential in this respect.

### Informing Further Development or Implementation

**Figure 2:**
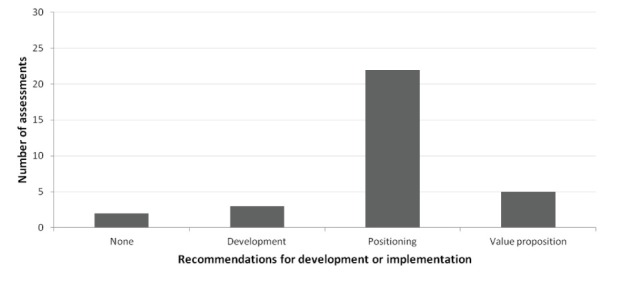
Overview of the Recommendations for Development or Implementation.

In 2 (6%) assessments no recommendations were provided on further development (Figure 2).

The other 30 (94%) assessments informed how the innovation should be further developed or implemented to enhance the cost-effectiveness of the innovation. Three assessments specifically focused on further development. One of these was a screening tool (#9), where the assessment clearly showed that the impact of the innovation could be increased by increasing specificity. This informed the developers that they should set their cut-off values in a way that allowed optimal specificity. Another was an innovation that aimed to reduce under- and overtreatment by improving patient compliance to medication (#11). During the assessment we found evidence that a lower dose did not result in disease progression. Hence, reducing under-treatment would result in high costs without yielding health benefits. Based on this information we recommended to develop the innovation in a way that the focus was on reducing overtreatment.

Most (69%) of the assessments showed how decisions on positioning of the innovation in the care pathway impacted its cost-effectiveness. Positioning could concern the target population or the place the innovation has in a care pathway, which could also be related. For example, for a diagnostic innovation for bladder cancer (#19) we modeled the potential consequences of implementation of the innovation before and after a standard diagnostic test. We found that for specific subgroups of patients most value for money was expected when implementing the innovation before the standard test, while for other subgroups most value for money could be achieved when the innovation was used after the standard test. These subgroups were based on symptoms of the disease, and thus the cost-effectiveness of the innovation could be maximized if the positioning of the innovation is tailored to the presence of these symptoms.

Another example was an add-on therapy for cancer treatment (#27). This could be added to the alternative (second-line) treatment to increase its effectiveness. We explored the potential health economic consequences of the innovation added to second-line treatment for 3 different implementation strategies: (1) as an alternative to first-line treatment, (2) as an alternative to second-line treatment without the innovation, and (3) as an alternative to first-line treatment only if this was unavailable due to delivery issues, which was a realistic problem. In addition, we explored the consequences of a strategy in which we added the innovation to second-line treatment with the aim to give patients half the treatment dose patients receive now. Each strategy thereby represented a possible positioning of the innovation in the care pathway. This analysis clarified the potential risks and benefits of each scenario, and therefore informed the decision on how to position the innovation. We found that it was not worthwhile to use the innovation to reduce the treatment dose, as this would only result in limited health benefit at increased costs. We also found that adding the innovation to second-line treatment for patients who are intolerant or do not respond to first-line treatment (strategy 2) has most potential value.

Five (16%) of the assessments resulted in recommendations on the innovation’s value proposition, ie, how the developers propose the innovation to add value to the standard of care. For example, we assessed the potential value of an innovation that aimed to reduce the time needed for a specific part of a procedure (#4). We found that the health benefit of reducing time for this part of the procedure was limited and uncertain. Hence, we advised the clients to shift the value proposition to a different procedure or aim, such as improved precision.

### Informing Further Research

**Figure 3:**
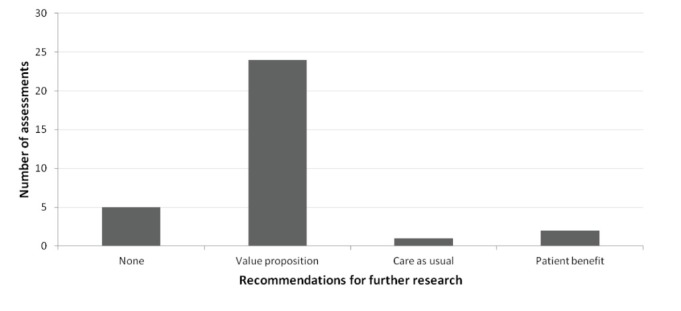
Overview of the Recommendations for Development or Implementation.

Most (75%) of the assessments recommended to perform empirical research to (further) validate the value proposition (Figure 3).

In these assessments we found that the innovation was *potentially* cost-effective. However, as for most innovations no or limited clinical evidence on their effectiveness was available, we recommended to empirically study this effectiveness to assess the *actual* cost-effectiveness of the innovation. For example, developers of an innovation claimed that the innovation could increase medication adherence (#12). The assessment showed that if this could be realized, this would result in considerable cost savings and health benefits. The recommended next step was therefore to study the percentage of adherence that could be increased, and its consequences. With the use of scenario and sensitivity analyses the assessments allowed for very specific recommendations on the target population, outcome measures and time horizon that should be used in future studies.

Also, specific recommendation on research into parts of the value proposition were provided. For example, for an innovative screening tool (#16) we found that the participation rate was considerably influencing its potential cost-effectiveness. Hence, it was important that studies investigated – next to accuracy of the tool – what a realistic participation rate would be. This might require different study designs, such as qualitative research, surveys or discrete choice analysis. For some innovations we showed that it was important not only to study whether an innovation works, but also to study whether this can result in improved health. It is often possible to study the relationship between the direct aim of the innovation (eg, reduced complications) and improved health, even before developing and studying the specific innovation. Since the cost-effectiveness of the innovation is dependent on the consequences of its aim (eg, the health gain through reducing complications), it was sometimes recommended to study these consequences before further developing the innovation and studying whether it works.

Related to this, one assessment (3%; #17) recommended to perform further research into the current care pathway, to better determine the effectiveness gap (ie, difference between current effectiveness and a hypothetical perfect situation). Here, the uncertainty about the effectiveness gap was found to be too high to draw valid conclusions on the potential cost-effectiveness of the assessment. The assessment revealed specifically which lacking information was influential and should thus be collected.

For the 7 innovations where some effectiveness data were already available, the assessments showed the importance of measuring for example patient-related outcomes, or performing better quality studies into effectiveness and/or societal impact. Two innovations (6%) with performance data – both diagnostic innovations – were deemed to have sufficient evidence on cost-effectiveness for widespread implementation.

In 2 other assessments (6%) that did not recommend further research, the recommendation was to further develop the innovation or change the value proposition, before conducting further research.

## Discussion

Our exploration of 32 early health economic assessments resulted in 3 main insights. First, we found that all 30 innovations could potentially become cost-effective. The assessments did provide insight in the size and uncertainty of the potential cost-effectiveness of the innovation in its intended context. Second, we found that the assessments were helpful in informing how the innovation could be further developed or should be positioned to maximize its value for money. Third, we found that the assessments were helpful in steering further research, with specific recommendations made for endpoints, types of studies and target populations.

In previous studies, Markiewicz et al^10^ and Chapman et al^11^ concluded that early health economic modeling is able to distinguish favorable and unfavorable innovations. Although we found differences between innovations in the magnitude of the potential cost-effectiveness, and the likelihood of reaching this potential, we were not able to dismiss any of the 30 innovations because they all were potentially cost-effective through improving health and/or saving costs. With our analyses we informed the clients on this magnitude and likelihood, as well as on how they can best continue development to maximize the cost-effectiveness of the innovation. Also, we found that commercial viability, or potential value of an innovation comprises more than whether it has the potential to become cost-effective compared with care as usual. Budget impact, meeting needs of patients or clinicians, risks, competing upcoming innovations and logistical issues may limit the commercial viability of an innovation, even if it is potentially cost-effective. Although these broader criteria were not quantified in the health economic models, these were encountered in the accompanying interviews and included in the assessments reports. For example, one innovation resulted in only limited cost savings per patient and no health benefit. However, as the innovation would be applied in a large patient population, and its expected costs are limited, it could result in large savings for society. For some innovations there was potential – theoretical – value, but it was deemed very difficult to achieve this value. Pursuing innovation would thus be risky as the probability that the innovation would become cost-effective was deemed low. Here, we advised developers to reconsider whether it was worth taking this risk.

It is known that there are many challenges for implementation of a health innovation.^1,17^ As Lehoux et al show, many of these challenges characterizing the later phases of development (eg, implementation or diffusion), are determined at a much earlier stage.^2^ We found that early health economic modeling can help to identify at least some of these challenges in an early stage, to allow for development and research to be tailored to address these potential barriers. One specific barrier that we encountered several times was that an innovation would result in reallocating care from one clinical specialty to another. Or, that a relatively cheap device could potentially obviate expensive surgery. However, the expensive surgery was reimbursed, whereas the device was not. As a result, use of the device would cost the hospital money, and while it could potentially result in considerable societal savings, these savings would not benefit the hospital. This makes it difficult to implement the device in the hospital. These examples emphasize the importance of understanding the (financial) context of the healthcare setting the innovation is going to be positioned in. Using stakeholder interviews to inform the health economic modeling assessment can help to yield important information about this context.^18^

Our study also has some limitations. First, our study is a retrospective analysis 32 early-stage health economic modeling assessments performed by a subsidiary company of our university hospital. Almost all assessments were commissioned by the developers of the innovation, and contain sensitive information about the innovation and its potential cost-effectiveness. To date, only 2 of the assessments were published.^19,20^ Due to the confidentiality of the information, we were limited in the amount of information we could provide on the assessments. Confidentiality is often an issue in early HTA, when commercial viability is assessed. However, we believe it is important to share experiences such as these to improve the use and methodology of early HTA.

Second, the innovations analyzed here represent a selected sample of innovations for which an early health economic modeling study was commissioned. This may have overestimated the potential cost-effectiveness, because developers of these innovations are sufficiently confident to commission an early economic modeling study. On the other hand, one could argue that it is an underestimation, because for the medical technologies with most potential value implementation in practice is easy and a health economic modeling study would not be commissioned. It is unclear if repeating this study in another setting would result in similar findings. However, we expect that headroom analysis, being a best-case and often unrealistic scenario, will hardly ever result in firm ‘no-go’ decisions. We encourage others to share similar empirical research on early health economic modeling, to corroborate or disprove our findings. Third, the innovations varied in their phases of development. It is questionable whether the assessments of the innovations that were in the market access phase can be defined as ‘early.’ Ijzerman et al define early HTA as “all methods used to inform industry and other stakeholders about the potential value of new medical products in development, including methods to quantify and manage uncertainty.”^5^ They add that this definition “includes early HTA of medical products just before and also at the early stages of clinical use, while accepting that product development can continue after regulatory approval”. With this addition, all of our assessments can be defined as ‘early.’ Fourth, we did not provide results of the assessments in terms of expected costs or effects, or cost-effectiveness estimates. Besides issues of confidentiality mentioned earlier, we feel that insight in potential consequences, and the relationship between assumptions and consequences were much more important than a point estimate. Also, the point estimate cannot be judged without the context of the analysis performed, the quality of the evidence underlying the assessment, the likelihood of an innovation reaching its potential and so on. Fifth, related to the quality of evidence, we addressed uncertainty surrounding care as usual only by means of deterministic sensitivity analyses. Ideally, we would have combined deterministic and probabilistic sensitivity analyses to best address the existing uncertainty and its interrelatedness.^14^ However, in this early stage it is difficult to quantify the (non-statistical) uncertainty surrounding the costs and effectiveness of the innovation in distributions. Probabilistic sensitivity analyses in this case can cause pseudo-certainty of the results generated and may therefore be misleading.^21^ For an audience without expertise in economic evaluation, we feel that scenario and threshold analyses, as well as deterministic sensitivity analyses, for example presented in tornado diagrams, are most informative in providing insight in the factors that most impacted the cost-effectiveness of the innovation. Sixth, while we clustered the different insights, these were not as black and white as presented here. Generally, assessments provided multiple recommendations and these may relate to different clusters. We here presented the most important insights, but these can also not be interpreted as being independent. For example, the value proposition is often dependent on the positioning of the innovation in the care pathway, and the recommendations for further research are strongly related to both the value proposition and positioning.

The current study focuses on the objective assessment of potential value for money of innovations. While we could not recommend *not* to pursue an innovation because all had some potential value, we subjectively could distinguish the more valuable from the less valuable innovations. Further research is needed to define objective criteria that help to prioritize innovations, such as the likelihood of an innovation closing the effectiveness gap, barriers for implementation, and existing uncertainty. In addition, it is interesting to see how these innovations proceed. First, the value of early economic modeling could be studied by exploring whether and how the clients of these assessments used the results. In a later stage, the early assessments could be validated when real-world evidence on the innovations becomes available.

Our results contribute to the understanding and interpretation of early health economic modeling, and potentially to the way such modeling studies are performed. For most of the innovations assessed here, evidence on their (cost-)effectiveness is not yet required to become widely used. If this evidence becomes mandatory in the new European Union Medical Device Regulation, the role, use and timing of early HTA may become more clear-cut.^22,23^ Ideally, early HTA informs and thereby integrates development, research and implementation decisions, and bridges the gap between development and use of promising innovations.^24^ Regardless of the availability and requirement of evidence, we believe that the ultimate goal of early HTA should be to accept the existing uncertainty, try to understand it, and make it part of our reasoning.^15^ By using scenario analysis (including the best-case headroom scenario) it is possible to explore what could be the consequences of an innovation if certain assumptions (eg, regarding positioning or effectiveness) are made. This provides insight in how to proceed, both in terms of development of the innovation and further research. This implies a shift away from traditional use of health economic modeling with the aim of estimating the exact cost-effectiveness of a technology, towards exploring what is needed for a technology to provide most value for money.

## Conclusion

Our exploration of 32 early health economic modeling assessments showed that the assessments provided insight in the size and uncertainty of the potential cost-effectiveness of an innovation in its intended context. Although we found differences between innovations in the magnitude of the potential cost-effectiveness, and the likelihood of reaching this potential, none of the assessments resulted in a firm ‘no-go’ recommendation because they all were potentially cost-effective by improving health and/or saving costs. The assessments did provide insight in how to proceed, both in terms of development and positioning of the innovation and further research, in order to maximize value for money.

## Acknowledgements

No funding was received for conducting this study. The individual assessments of innovations were funded by the commissioners of the assessments, generally small to medium enterprises. All assessments were independently and objectively performed by a not-for-profit company. We gratefully acknowledge all researchers who performed the assessments, Sabine Mulders, and the commissioners of the assessments who have made this study possible. The participants of lolaHESG 2018 are acknowledged for their constructive feedback to the paper.

## Ethical issues

This study did not use data collected from human subjects, ethics approval therefore was not required.

## Competing interests

The underlying assessments on which this aggregate analysis was based were performed independently of the study reported here. These were funded by the commissioners of the assessments, generally small to medium enterprises with a financial interest in the outcome. All assessments were independently and objectively performed by a not-for-profit company with no proprietary or financial interest in the outcome. The authors declare that they have no conflicts of interest regarding the study reported in this manuscript.

## Authors’ contributions

All co-authors have made a substantial contribution to the manuscript; they revised it critically for important content and approved the final version.

## Authors’ affiliations

^1^Department for Health Evidence, Radboud Institute for Health Sciences, Radboudumc, Nijmegen, The Netherlands. ^2^Medvalue, Radboudumc, Nijmegen, The Netherlands. ^3^Department for Health Evidence, Donders Institute for Brain, Cognition and Behaviour, Radboudumc, Nijmegen, The Netherlands. ^4^Department of Operating Rooms, Radboud Institute for Health Sciences, Radboudumc, Nijmegen, The Netherlands.

## Supplementary files


Supplementary file 1. Overview of the Innovations and Assessments.

Supplementary file 2. Example of a Model-Based Health Economic Assessment.

## Key messages

**Implications for policy makers**

- Early health economic modeling provides insight in potential cost-effectiveness of a healthcare innovation in its intended context, and the associated uncertainty.
- This can be applied before and during the development of an innovation.
- Of the 32 modeling assessments that were retrospectively analyzed, none resulted in a firm ‘no-go’ recommendation because they all could potentially become cost-effective by improving health and/or saving costs.
- The assessments did provide insights in how to proceed, both in terms of development and positioning of the innovation and further research, in order to maximize value for money.
- This implies a shift away from traditional use of health economic modeling with the aim of estimating the exact cost-effectiveness of a technology, towards exploring what is needed for a technology to provide most value for money.

**Implications for public**

The value an innovative health technology may ultimately bring to healthcare systems is defined at an early stage. Health economic modeling can be used in this early stage to synthesize evidence on the current standard of care and compare this with a hypothetical care pathway that includes the technology. This provides insight in the potential value for money the innovation can bring in its intended context, as well as the associated uncertainty, and informs further research and development. None of the 32 assessments we retrospectively explored resulted in a firm ‘no-go’ recommendation, but recommendations could be provided on further research and development in order to maximize value for money.
