# Supplementary material for: Problems and Promises of Health Technologies: The Role of Early Health Economic Modeling
Source: Int J Health Policy Manag. 2019 Jun 22;8(10):575–82. doi: 10.15171/ijhpm.2019.36 (PMC6819627; doi:10.15171/ijhpm.2019.36)
Supplement: Supplementary file 1 — Overview of the Innovations and Assessments. [file ijhpm-8-575-s001.pdf]

Supplementary file 1. Overview of the innovations and assessments

#	Type of innovation	Clinical area	Purported impact on clinical pathway[1]	Clinical data on innovation available?	Type of model[2]	Time horizon	Analyses	Primary outcome[3]
1	Diagnosis	Obstetrics	Partial substitute	No	DT	Short term	Scenario	Clinical
2	Treatment	Oncology	Substitute	Yes, safety study	STM	Lifetime	CEA, scenarios	QALYs
3	Screening	Obstetrics	Add-on	No	DT	Short term	Headroom, threshold, scenario	Clinical
4	Treatment	Nephrology	Substitute	No	STM	5 years	Scenarios	Clinical
5	Treatment	Oncology	Substitute	No	DT	Short term	Scenarios	Clinical
6	Diagnosis	Oncology	Partial substitute	Yes, accuracy study	DT+STM	10 years	CEA, scenarios	QALYs
7	Treatment	Anesthesiology	Add-on	No	DT	Short term	Headroom, threshold, scenarios	Clinical
8	Diagnosis	Pulmonology	Add-on	No	DT+STM	Lifetime	Headroom, scenarios	QALYs
9	Screening	Oncology	Add-on	No	DT+STM	20 years	Threshold, scenarios	QALYs
10	Diagnosis	Critical care	Add-on	No	DT+STM	10 years	Headroom, scenarios	QALYs
11	Treatment	Hematology	Add-on	No	DT	1 year	Headroom, scenarios	Clinical
12	Treatment	Hematology	Add-on	No	STM	25 years	Headroom, scenarios	QALY
13	Diagnosis	Oncology	Partial substitute	Yes, accuracy study	DT+STM	18 years	CEA	QALY
14	Treatment	Endocrinology	Add-on	No	STM	Lifetime	Scenarios	QALY
15	Treatment	Endocrinology	Substitute	No	STM	Lifetime	Scenarios	QALYs
16a	Screening	Oncology	Partial substitute	Yes, accuracy study	DT+STM	Lifetime	CEA, scenarios	Lifeyears
16b	*Same innovation as 16a*		Partial substitute	Yes	DT+STM	Lifetime	Scenarios	Lifeyears
17	Diagnosis	Obstetrics	Partial substitute	No	DT+STM	Lifetime	Headroom, threshold	QALYs
18	Treatment	Orthopedics	Add-on	No	DT	Short-term	Headroom, threshold	Clinical
19a	Diagnosis	Oncology	Partial substitute	Yes, accuracy study	DT+STM	Lifetime	CEA, scenarios	QALYs
19b	*Same innovation as 19a*		Add-on		DT + STM	Lifetime	Scenarios	QALYs
20	Diagnosis	Pulmonology	Add-on	No	STM	Lifetime	Scenarios	QALYs

21	Screening	Cardiology	Add-on	No	DT + STM	Lifetime	Scenarios	QALYs
22	Treatment	Oncology	Add-on	No	DT + STM	Lifetime	Scenarios, threshold	QALYs
23	Treatment	Oncology	Add-on	Yes, intermediate outcome	DT	Lifetime	CEA, threshold	QALYs
24	Diagnosis	Oncology	Partial substitute	Yes, accuracy study	DT + STM	Lifetime	CEA, scenarios	QALYs
25	Diagnosis	Cardiology	Add-on	No	DT + STM	Lifetime	Headroom, scenarios, threshold	QALYs
26	Treatment	Oncology	Add-on	No	DT + STM	Lifetime	Headroom, scenarios	QALYs
27	Treatment	Oncology	Add-on	No	DT + STM	Lifetime	Scenarios	QALYs
28	Screening	Geriatrics	Add-on	No	STM	1 year	Scenarios, threshold	QALYs
29	Treatment	Surgery	Add-on	No	DT	Lifetime	Threshold, scenarios	QALYs
30	Diagnosis	Oncology	Add-on/substitute	No	DT + STM	20 years	Threshold, scenarios	QALYs

[1] The innovation could be added to current practice (add-on), substitute current practice (substitute) or be added with the aim to prevent part of current practice (partial substitute). An example of the latter is a diagnostic test that is implemented to reduce the need for an existing (more invasive) test.

[2] In all assessments the innovation was compared with care as usual, adopting a Dutch healthcare perspective, except for three assessments that adopted a Canadian healthcare (#2), a United States Medicare (#24) and a Norwegian healthcare (#28) perspective.

[3] In addition to this primary outcome, in all assessments costs were evaluated.

Abbreviations: DT=Decision Tree; STM=State Transition (Markov) Model.
