# Supplementary material for: Problems and Promises of Health Technologies: The Role of Early Health Economic Modeling
Source: Int J Health Policy Manag. 2019 Jun 22;8(10):575–82. doi: 10.15171/ijhpm.2019.36 (PMC6819627; doi:10.15171/ijhpm.2019.36)
Supplement: Supplementary file 2 — Example of a Model-Based Health Economic Assessment. [file ijhpm-8-575-s002.pdf]

Supplementary file 2. Example of a Model-Based Health Economic Assessment

Context (disease, problem, value proposition)

For one of the assessments, the innovation was an e-health technology that was not yet developed (#12). Due to improved treatment, the prevalence of chronic myeloid leukemia is steadily rising worldwide, resulting in an increase in costs and healthcare utilization. The problem addressed by this innovation is that physicians' adherence to the international guidelines, as well as patient adherence to treatment in chronic myeloid leukemia is suboptimal. The value proposition of the e-health technology was that improved physician and/or patient adherence will gain health and save costs.

Model

A state transition model was constructed to quantify the potential impact on health benefit and costs of (1) improving patient adherence to treatment (tyrosine kinase inhibitors) and (2) improving guideline adherence by physicians. Patient and physician adherence were modeled separately. This allowed us to inform the developers for which type of non-adherence improvement was - in theory - most valuable. First, evidence on care as usual was synthesized to estimate the average healthcare costs and QALYs over 25 years. Details on the model and input parameters are described elsewhere (Ector et al, 2019).

Headroom analysis

In a headroom analysis, the current situation was compared to a perfect situation, where adherence was 100% for either the physician or patient. The difference in costs and QALYs was calculated between the current and perfect situation. In the headroom analysis no costs are assigned to the perfect strategy, which implies that the difference in costs can be interpreted as the costs that could be made for the technology in order to improve adherence without making the care pathway more expensive.

Scenario analysis

If the expected costs or effectiveness of a technology are unknown, scenario analysis can be used to calculate 'what if' statements. For example, we could have estimated the difference in costs and QALYs if the technology increased adherence by 50%. In this example, the impact

on costs and QALYs was calculated per percentage point increase in adherence. This way, the reader can calculate the cost-effectiveness for any possible scenario of increased adherence.

Threshold analysis (not performed in this case)

In this example, the technology was not yet being developed, and its expected costs and effectiveness were unknown. Had the expected costs of the technology been known, one could have calculated the increase in percentage points adherence needed for the technology to become cost-effective. Or, had the expected increase in adherence been known, one could have calculated the maximum costs of the technology in order to become cost-effective.

Cost-effectiveness analysis (not performed in this case)

In case the technology was developed and tested in clinical practice, a more traditional cost-effectiveness analysis could be performed. For example, if in a study researchers found that the e-health technology increased adherence by 20%, this result could be incorporated in the model to estimate the cost-effectiveness of the technology. In this phase, the model is used to translate an intermediate outcome (e.g. adherence) into costs and QALYs.

Sensitivity analysis

Headroom, scenario and threshold analysis are ways to deal with uncertainty surrounding the innovation, on which much uncertainty exists in an early stage of development. However, there is also uncertainty on the current care pathway. In the assessments this is dealt with using deterministic sensitivity analyses. In this example we investigated the impact of the price of TKIs on the headroom, as well as for example the impact of the relationship between adherence and molecular response on the headroom.

Reference

Ector GICG, Govers TM, Westerweel PE, Grutters JPC, Blijlevens NMA. The potential health gain and cost savings of improving adherence in chronic myeloid leukemia. Leuk Lymphoma. 2019 Jan 22:1-8. doi: 10.1080/10428194.2018.1535113.
